# Features of low-dose CT-detected lung nodules: individuals who never smoked vs. who smoke(d) in a Chinese general population

**DOI:** 10.1007/s00330-026-12517-4

**Published:** 2026-04-17

**Authors:** Yifei Mao, Monique D. Dorrius, Grigory Sidorenkov, Marcel van Tuinen, Xiaofei Yang, Xiaonan Cui, Zhaoxiang Ye, Rozemarijn Vliegenthart, Geertruida H. de Bock, Marjolein A. Heuvelmans

**Affiliations:** 1 Department of Epidemiology, University Medical Center Groningen, University of Groningen, Groningen, The Netherlands; 2 Radiology, University Medical Center Groningen, University of Groningen, Groningen, The Netherlands; 3 Department of Radiology, Tianjin Medical University Cancer Institute and Hospital, National Clinical Research Centre for Cancer, Key Laboratory of Cancer Prevention and Therapy, Tianjin’s Clinical Research Center for Cancer, Tianjin, China; 4 Department of Respiratory Medicine, Amsterdam University Medical Center, Amsterdam, The Netherlands; 5 Institute for Diagnostic Accuracy, Groningen, The Netherlands

**Keywords:** Tomography, X-ray computed, Multiple pulmonary nodules/diagnostic imaging, Lung neoplasms/diagnostic imaging, Tobacco smoking/adverse effects

## Abstract

**Objectives:**

To evaluate and compare low-dose CT (LDCT)-defined pulmonary nodule features between individuals who never smoked and who smoke(d) in a Chinese general population.

**Materials and methods:**

This study included 2033 participants from the Nelcin-B3 cohort who underwent baseline LDCT. Trained radiologists reviewed each CT scan and assessed nodule CT features, including nodule density, size, location, shape, edge, attachment type, calcification and perifissural nodules (PFNs). Multilevel logistic regression (adjusted for age and sex) was performed to evaluate the relationship between nodule CT features and smoking status, accounting for nodule clustering within participants.

**Results:**

Overall, 36.7% (746/2033) of participants had at least one ≥ 30.0 mm^3^ lung nodule (individuals who never smoked vs. who smoke(d): 33.6% vs. 42.4%, *p* < 0.001), with 1267 nodules were registered. Among those with nodules, the mean number of nodules per person was 1.6 (724/444) in individuals who never smoked, and 1.8 (543/302) in individuals who smoke(d) (*p* = 0.02). Individuals who never smoked more often had a single nodule compared to individuals who smoke(d) (67.3% vs. 58.3%, *p* = 0.008). No significant differences were observed in nodule CT features (*p* > 0.05), including nodule size, location, and morphology, between smoking groups.

**Conclusion:**

In the Chinese general population, smoking status was associated with lung nodule prevalence and nodule number, but not with key nodule CT features. These findings suggest that in Asian populations, nodule characteristics may be influenced by factors beyond smoking, underscoring the need for population-specific risk stratification strategies in lung nodule assessment.

**Key Points:**

***Question*** *Lung nodule risk stratification relies on their CT features, yet no Asian studies have comprehensively examined these features between individuals who never smoked and who smoke(d)*.

***Findings*** *CT features of lung nodules (e.g., size, location, morphology) showed no significant differences between smoking groups in a Chinese general population, despite observed differences in nodule prevalence and count*.

***Clinical relevance**** Relying solely on smoking history may underestimate the malignancy risk of lung nodules in Asian populations, emphasizing the need for population-specific risk stratification strategies*.

**Graphical Abstract:**

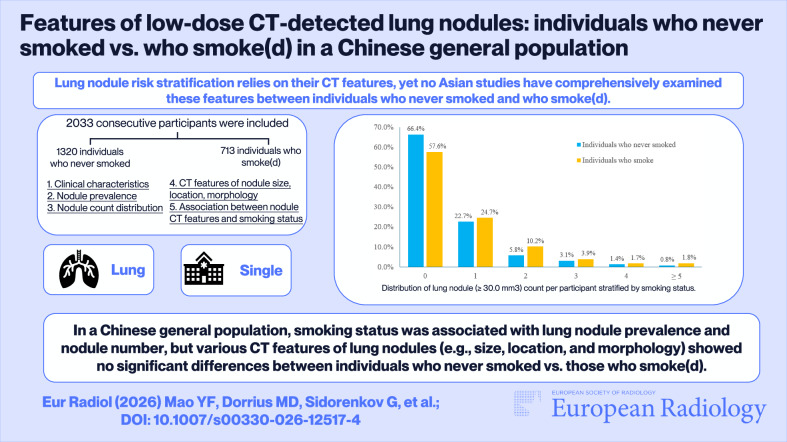

## Introduction

Lung nodules, defined as round or irregular opacities located within the lung parenchyma, measuring < 3 cm in maximum diameter, are increasingly detected due to the widespread use of chest computed tomography (CT) [1, 2]. Over 95% of lung nodules incidentally found through CT are considered benign [3]. In general, the malignancy risk of a lung nodule is strongly associated with its features on CT images, such as nodule density, size, location, and calcification [4–7]. The Fleischner Society has issued management guidelines based on these imaging features and clinical risk factors, including smoking history, age, and family history. The most recent update in 2017 introduced a risk stratification model incorporating both CT and clinical characteristics [7].

While smoking is the primary risk factor for lung cancer, recent findings suggest only minor differences in CT features of lung nodules between individuals who never smoked and those who currently smoke or previously smoked (hereafter referred to as “individuals who smoke(d)”). The study by Cai et al, conducted based on Fleischner Society 2017 guidelines, found that while irregular nodule edge and shape were less common in individuals who never smoked, there were no significant differences between individuals who never smoked and who smoke(d) in other important features, such as nodule size and location [8]. The findings by Cai et al focused on solid nodules in a Dutch general population aged 45–60 years. Therefore, the generalizability of these findings to an Asian population can be questioned. Notably, compared to Western individuals who never smoked, there is a higher prevalence of lung nodules among individuals who never smoked in East Asia [9, 10], and a higher burden of infectious lung diseases such as tuberculosis in some Asian countries [11]. Several Asian studies have examined CT features of lung nodules in individuals who never smoked [12–14]. However, they either analyzed only a few features or did not provide direct comparisons between individuals who never smoked and who smoke(d). Thus far, it remains unclear how nodule CT features differ across individuals with different smoking status in an Asian population, and a comprehensive evaluation of multiple malignancy-associated CT features in this context is still lacking.

This study aims to compare CT features of lung nodules between individuals who never smoked and who smoke(d) in a general Chinese population, to better characterize the association between smoking exposure and the distribution of established malignancy-associated imaging features in a real-world screening setting.

## Materials and methods

### Study sample selection

**Fig. 1:**
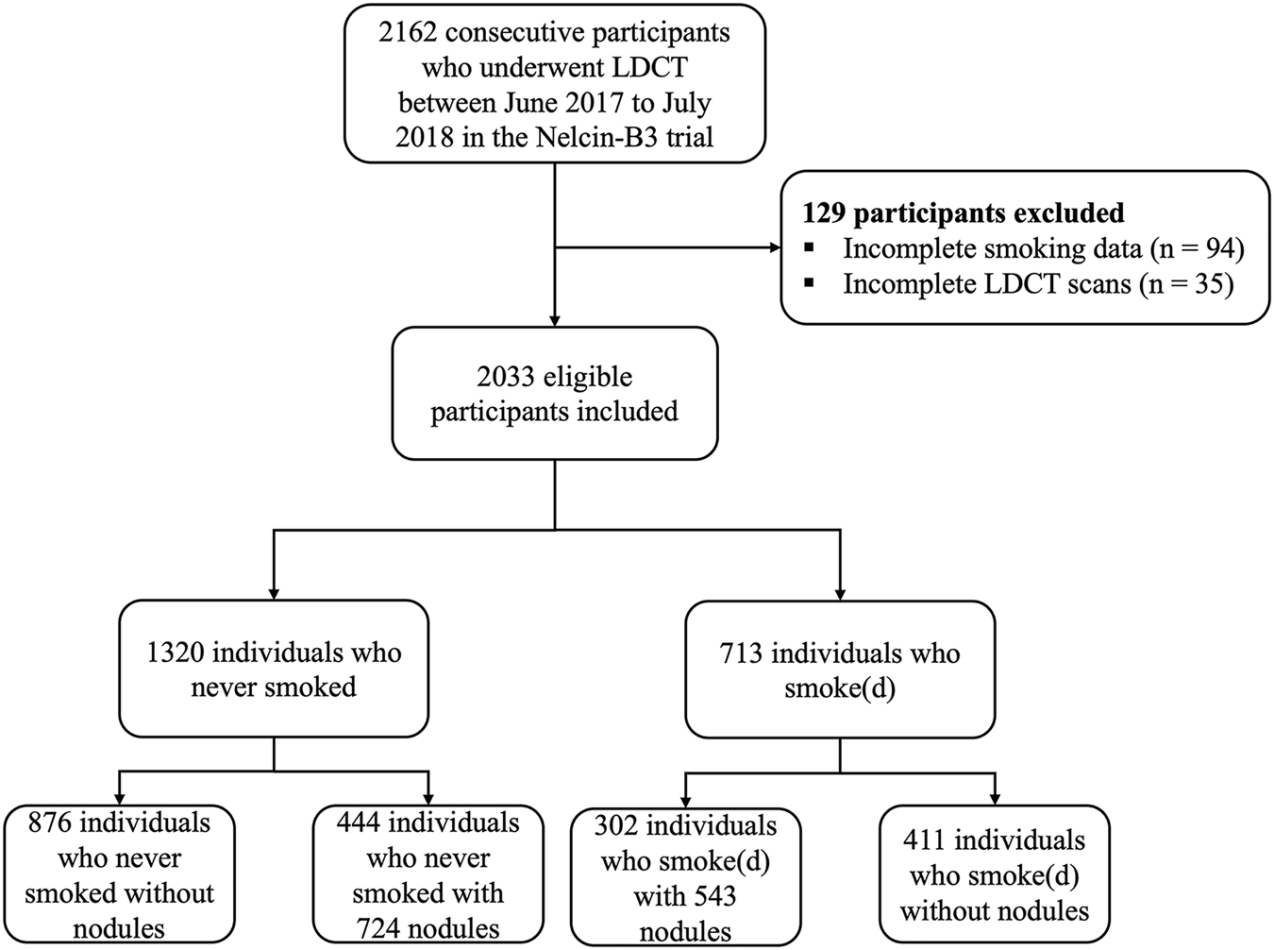
Flowchart of study design

The present study is a retrospective and secondary analysis of the Netherlands and China Big 3 diseases (Nelcin-B3) trial, which was designed to facilitate the early diagnosis of lung cancer, cardiovascular disease, and COPD (registration number: NCT03992833). The detailed study design of the NELCIN-B3 study and part of the baseline results have been reported previously [15–17]. As part of the Nelcin-B3 trial, at the Tianjin Medical University Cancer Institute and Hospital, individuals who had lived in Tianjin for at least 3 years, aged 40–74 years, with no self-reported history of cancer, were invited to undergo LDCT screening. All participants signed the informed consent. For the present analysis, consecutive participants with available baseline LDCT examinations between June 2017 and July 2018 were initially included. Exclusion criteria included (1) incomplete data on smoking status and (2) incomplete LDCT scans (see Fig. 1).

### Clinical data collection and definitions

Face-to-face questionnaire interviews were conducted by trained interviewers to obtain demographic data (age, sex) and smoking history for all participants (Table S1). Smoking history included smoking status (individuals who never smoked, individuals who smoke(d)), pack-years, duration since smoking cessation, and second-hand smoking exposure. Second-hand smoking was defined as inhalation of tobacco smoke produced by other people for ≥ 15 min on one or more days per week in indoor environments. Clinical data collection and CT scans were performed on the same day.

### CT scan acquisition

LDCT scans were acquired using a 128-detector row CT scanner (Somatom Definition AS 128, Siemens Healthineers) with a tube voltage of 120 kVp, a reference tube current of 35 mAs, a maximum volume CT dose index (CTDIvol) of 2 mGy, and an estimated effective dose of ≤ 2 mSv. The images were reconstructed using a medium-soft kernel (D45f) and a hard kernel (B80f) at a slice thickness of 1.0 mm and an increment of 0.7 mm.

### Image assessment and nodule features

Each LDCT scan was reviewed by one of three radiologists (M.D., X.Y., and Y.M.), who were blinded to clinical information. Two junior radiologists (X.Y. with 5 years of experience in chest CT reading, and Y.M. with 4 years of experience in chest CT reading) received training in the detection and characterization of lung nodules from a senior radiologist (M.D.), who had over 10 years of experience in chest CT reading. All readers used a standardized reading protocol. The window settings for lung (width, 1200 HU; level, −500 HU) and mediastinum (width, 320 HU; level, 35 HU) were used for scan interpretation. Lung nodules were detected manually by readers using maximum intensity projection (MIP) reconstruction under D45f kernel with 10-mm slice thickness. No computer-aided detection system was used for automatic nodule detection. Nodule size, including both diameter and volume, was measured using semi-automatic segmentation software (MM Oncology, Syngo.via VB30, Siemens Healthineers) under the B80f kernel with 1-mm slice thickness. Readers were instructed to detect and annotate nodules with a total volume of ≥ 30.0 mm^3^ (approximately 3 mm diameter). If the LDCT scan had multiple nodules, the 10 largest nodules were registered in the nodule management system.

For registered nodules, readers determined their CT features, including nodule density (solid, part-solid, or pure ground-glass nodule), size, location, shape, edge, attachment type, calcification and perifissural nodules (PFNs). Nodule size was categorized into three groups: < 6 mm, 6–8 mm, and > 8 mm based on the average diameter (the average of maximum axial and maximum orthogonal diameter), or into three groups: < 100 mm^3^, 100–250 mm^3^ and > 250 mm^3^ based on the total volume, following Fleischner Society Guidelines [7]. Rounding mean diameter to the nearest millimeter was applied. Location included location in lung lobe (upper lobe [left, or right], middle [right] or lower lobe [left or right]) and location within lung (central or peripheral). Shape was classified as regular (spherical, oval, triangular or polygonal) or irregular. Edge was defined as either smooth or non-smooth (lobulated, spiculated, irregular, and fuzzy). Attachment, which refers to a nodule’s relationship to surrounding tissues, was classified into four types, as follows: (1) intraparenchymal (no attachment with other tissues), (2) pleura-attached (attached to the pleural wall), (3) vessel-attached (attached to a vessel), and (4) fissure-attached (attached to a fissure). The presence of nodule calcification was classified as yes (fully, centrally, popcorn, rim or other calcification types) or no. The presence of PFNs was classified as yes (typical or atypical PFN) or no (non-PFN) [4].

### Statistical analysis

All statistical analyses were conducted using SPSS for Windows (version 25.0), with *p* < 0.05 treated as statistically significant. Categorical variables were described by absolute frequencies and percentages, and continuous variables by medians and interquartile ranges (IQR) for non-normally distributed data. Normality was tested by the Shapiro–Wilk test. Participants or nodules were categorized by smoking status (definition in Table S1). The prevalence of lung nodules (≥ 30 mm^3^) in our study population was examined (see Supplementary Appendix 1).

First, a descriptive analysis was performed for the clinical characteristics of participants with nodules (*n* = 746). To examine between-group differences, the Chi-square test was used for categorical variables, and the Mann–Whitney U test was used for non-normally distributed continuous variables. Second, the number of LDCT-detected lung nodules per participant was assessed and classified into five groups: 1 nodule, 2 nodules, 3 nodules, 4 nodules, and ≥ 5 nodules. Differences in nodule count category prevalence between individuals who never smoked and who smoke(d) were evaluated by Chi-square test. Third, descriptive analyses of nodule CT features were performed at a per-nodule level (*n* = 1267). Nodules from the same person share common physiological, genetic, and environmental factors. Thus, these nodules tend to be more similar to each other than to those from different individuals, leading to a clustering effect. If unaccounted for, this effect may lead to an overestimation of the association between smoking status and nodule CT features. To address this, in the final analysis, a multilevel logistic regression with nodules clustered within participants was used to test the association between categorized nodule CT features and smoking status (binary data; individuals who never smoked or who smoke(d)) using the generalized linear mixed model. Sex and age (continuous data) were used as adjustment variables. Adjusted odds ratios (aORs) and corresponding 95% confidence intervals (CIs) were determined for each CT feature of lung nodules.

## Results

### Participants included in the analysis

Of 2162 participants with baseline LDCT, 129 were excluded due to missing smoking status or incomplete LDCT scans, yielding a final sample of 2033 participants (Fig. 1). Of them, 53.1% were female, and 35.1% were individuals who smoke(d) (Table S2). In total, 746 (36.7%) had at least one ≥ 30.0 mm^3^ lung nodule, and 1267 nodules ≥ 30.0 mm^3^ were registered. Individuals who smoke(d) showed a higher prevalence of lung nodules than those who never smoked (42.4% vs. 33.6%, *p* < 0.001). Age-specific prevalence of lung nodules is shown in Fig. S1 and Supplementary Appendix 2.

### Characteristics of participants with nodules

Among participants with nodules (*n* = 746), individuals who never smoked were younger (median age: 62 vs. 64), and less frequently male (24.2% vs. 97.0%) than those who smoke(d) (Table 1). Of the 302 individuals who smoke(d) with nodules, 59.3% had a smoking history of ≥ 20 pack-years, and 15.9% had quit smoking more than 10 years. In addition, second-hand smoking exposure was less frequent in individuals who never smoked (34.9%) compared to individuals who smoke(d) (59.3%).

**Table 1:** Characteristics of 746 participants with at least one lung nodule, overall and stratified by smoking status

Variables	Overall (*n* = 746)	Individuals who never smoked (*n* = 444)	Individuals who smoke(d) (*n* = 302)
Age (years), median (IQR)*	63 (59–67)	62 (57–66)	64 (60–68)
Sex			
Female	345 (46.2%)	336 (75.7%)	9 (3.0%)
Male	401 (53.8%)	108 (24.2%)	293 (97.0%)
Pack-years, median (IQR)*	-	-	20 (12–31)
Pack-years			
< 20	-	-	118 (39.1%)
≥ 20	-	-	179 (59.3%)
Missing	-	-	5 (1.7%)
Smoking cessation (years)			
< 10	-	-	253 (83.8%)
≥ 10	-	-	48 (15.9%)
Missing	-	-	1 (0.3%)
Second-hand smoking (years)			
No	410 (55.0%)	288 (64.9%)	122 (40.4%)
Yes, < 10	11 (1.5%)	4 (0.9%)	7 (2.3%)
Yes, ≥ 10	323 (43.3%)	151 (34.0%)	172 (57.0%)
Missing	2 (0.3%)	1 (0.2%)	1 (0.3%)

* Non-normally distributed data are medians with interquartile ranges in parentheses

### Lung nodule count per person

**Fig. 2:**
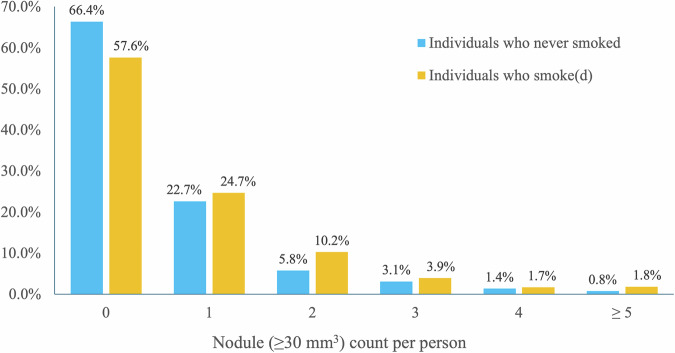
Distribution of lung nodule (≥ 30.0 mm^3^) count per participant, stratified by smoking status

Among participants with nodules, most (63.9%, 477/746) had a single nodule, 150 (20.1%) had two, 68 (9.1%) had three, 30 (3.8%) had four, and 23 (3.1%) had five or more nodules. Overall, the mean number of nodules per person was 1.6 (724 nodules/444 participants) for individuals who never smoked, and 1.8 (543/302) for those who smoke(d) (*p* = 0.02). Individuals who never smoked more frequently had a single nodule compared to individuals who smoke(d) (67.3% vs. 58.3%, *p* = 0.008), while individuals who smoke(d) more often had two nodules (24.2% vs. 17.1%, *p* = 0.011) than individuals who never smoked (Fig. 2). In addition, the proportions of participants having three (9.2% vs. 9.3%), four (4.1% vs. 4.0%), and ≥ 5 nodules (2.3% vs. 4.3%) were comparable between individuals who never smoked and who smoke(d) (*p* > 0.05).

### CT features of lung nodules

Of 1267 lung nodules identified in this study, the majority were solid (92.0%), especially in individuals who smoke(d) (96.0% vs. 89.1%, *p* < 0.001) (Table 2). Subsolid nodules (part-solid and non-solid) were more frequently detected in individuals who never smoked (10.9% vs. 4.0%, *p* < 0.001).

**Table 2:** Features of low-dose CT-detected lung nodules (*n* = 1267), stratified by smoking status

Variables	Overall (*n* = 1267)	Nodules in individuals who never smoked (*n* = 724)	Nodules in individuals who smoke(d) (*n* = 543)	*p*-value^#^
Nodule density				**< 0.001**
Solid nodule	1166 (92.0%)	645 (89.1%)	521 (96.0%)	
Part-solid nodule	27 (2.1%)	19 (2.6%)	8 (1.5%)	
Pure ground-glass nodule	74 (5.8%)	60 (8.3%)	14 (2.6%)	
Nodule size				
Average diameter, mm, median*	5.6 (4.9–6.8)	5.6 (4.8–6.8)	5.7 (4.9–6.7)	0.60
Average diameter, mm				0.84
< 6	749 (59.1%)	433 (59.8%)	316 (58.2%)	
6–8	339 (26.8%)	191 (26.4%)	148 (27.3%)	
> 8	179 (14.1%)	100 (13.8%)	79 (14.6%)	
Total volume, mm^3^, median*	59.0 (40.0–102.0)	59.0 (40.0–101.5)	58.0 (40.0–103.0)	0.96
Total volume, mm^3^				0.69
< 100	936 (73.9%)	535 (73.9%)	401 (73.9%)	
100–250	227 (17.9%)	126 (17.4%)	101 (18.6%)	
> 250	104 (8.2%)	63 (8.7%)	41 (7.6%)	
Nodule location				
Location within lung				**0.03**
Central	166 (13.1%)	82 (11.3%)	84 (15.5%)	
Peripheral	1101 (86.9%)	642 (88.7%)	459 (84.5%)	
Location in lobe				0.96
Upper lobe	491 (38.8%)	281 (38.8%)	210 (38.7%)	
Lower/middle lobe	776 (61.2%)	443 (61.2%)	333 (61.3%)	
Nodule shape				**0.02**
Regular	1156 (91.2%)	649 (89.6%)	507 (93.4%)	
Irregular	111 (8.8%)	75 (10.4%)	36 (6.6%)	
Nodule edge				**0.04**
Smooth	1068 (84.3%)	597 (82.5%)	471 (86.7%)	
Non-smooth	199 (15.7%)	127 (17.5%)	72 (13.3%)	
Nodule calcification				0.45
No	1002 (79.1%)	578 (79.8%)	424 (78.1%)	
Yes	265 (20.9%)	146 (20.2%)	119 (21.9%)	
Perifissural nodule (PFN)				0.69
No	1033 (81.5%)	593 (81.9%)	440 (81.0%)	
Typical/atypical PFN	234 (18.5%)	131 (18.1%)	103 (19.0%)	
Nodule attachment				0.50
Intraparenchymal	517 (40.8%)	296 (40.9%)	221 (40.7%)	
Pleura-attached	344 (27.2%)	204 (28.2%)	140 (25.8%)	
Fissure-attached	208 (16.4%)	109 | (15.1%)	99 (18.2%)	
Vessel-attached	198 (15.6%)	115 (15.9%)	83 (15.3%)	

Unless otherwise specified, data is numbers of participants with percentages in parentheses

* Non-normally distributed data are medians with interquartile ranges in parentheses

^#^ Our conclusion was based on participant-level analysis, not these *p*-values for nodule-level comparisons. Bold values indicate statistical significance (*p* < 0.05).

Median diameters and volumes were 5.6 mm (IQR: 4.8–6.8 mm) and 59.0 mm^3^ (IQR: 40.0–101.5 mm^3^) for individuals who never smoked, and 5.7 mm (4.9–6.7 mm) and 58.0 mm^3^ (40.0–103.0 mm^3^) for individuals who smoke(d), respectively (Table 2). At the per-nodule level, nodule size distribution was similar between individuals who never smoked and who smoke(d), with over 50% of nodules being small, measuring between 3.0 and 5.9 mm in average diameter (never-smokers: 59.8%; ever-smokers: 58.2%) and over 70% measuring 30–100 mm^3^ in total volume (73.9% in both groups). At the per-participant level, multilevel analyses adjusted for age and sex also revealed no significant association between nodule size and smoking status, whether measured by average diameter or total volume (Table 3).

**Table 3:** Association between smoking status and nodule CT features (univariate multilevel analyses adjusted for age and sex)

Variables	Individuals who never smoked compared to individuals who smoke(d)	
	Adjusted OR (95% CI)*	*p*-value
Nodule size		
Average diameter, mm		
< 6	1 (ref)	
6–8	0.99 (0.59–1.69)	0.98
> 8	1.19 (0.60–2.36)	0.61
Total volume, mm^3^		
< 100	1 (ref)	
100–250	1.08 (0.60–1.96)	0.8
> 250	1.68 (0.70–4.00)	0.24
Nodule location		
Location within lung		
Central	1 (ref)	
Peripheral	1.31 (0.68–2.53)	0.42
Location in lobe		
Upper lobe	1 (ref)	
Lower/middle lobe	1.43 (0.87–2.34)	0.16
Nodule shape		
Regular	1 (ref)	
Irregular	1.39 (0.60–3.23)	0.44
Nodule edge		
Smooth	1 (ref)	
Non-smooth	0.99 (0.51–1.91)	0.97
Nodule calcification		
No	1 (ref)	
Yes	0.55 (0.30–1.04)	0.06
Perifissural nodule (PFN)		
No	1 (ref)	
Typical/atypical PFN	1.00 (0.56–1.79)	0.99
Nodule attachment		
Intraparenchymal	1 (ref)	
Pleura-attached	1.13 (0.63–2.02)	0.68
Fissure-attached	1.20 (0.63–2.30)	0.58
Vessel-attached	1.14 (0.59–2.21)	0.70

Due to the small numbers of part-solid and non-solid nodules, the multilevel analysis was not performed for nodule density (solid, part-solid, and non-solid)

*OR* odds ratio, *CI* confidence interval

* Odd ratio is adjusted by age and sex

Regarding nodule location, no significant differences were observed when comparing individuals who never smoked to those who smoke(d) (*p* > 0.05 for both location in lung and lobe distribution) (Table 3). At the per-nodule level, the proportion of lower or middle lobe location was nearly identical (61.2% vs. 61.3%) between groups (Table 2). Most nodules were found in the peripheral lung (never-smokers: 88.7%; ever-smokers: 84.5%).

Morphological features were largely consistent between groups. Specifically, at the per-nodule level, most nodules in both groups had a regular shape (individuals who never smoked: 93.4%; who smoke(d): 89.6%) and smooth edge (individuals who never smoked: 86.7%; who smoke(d): 82.5%) (Table 2). At the participant level, multilevel analyses revealed no significant differences in these features (all *p* > 0.05) (Table 3). Similarly, the percentages of calcified nodules (20.2% vs. 21.9%) and PFNs (18.1% vs. 19.0%) were comparable between smoking groups at the nodule level. At the participant level, no significant associations were observed between the presence of nodule calcification (*p* = 0.06) and smoking status after adjusting for age and sex, although a lower aOR (0.55, 95% CI: 0.33–1.04) was observed in individuals who never smoked. No significant association was observed for PFNs (*p* = 0.99). Regarding nodule attachment, intraparenchymal nodules were most common (40.8%), followed by pleura-attached (27.2%), fissure-attached (16.4%), and vessel-attached (15.6%) nodules. Multilevel analysis revealed no significant difference in attachment types between groups (all aORs ≈1.1–1.2, *p* > 0.5). For the multilevel analyses adjusted additionally by second-hand smoking exposure, see Table S3. For the multilevel analyses without former smokers, see Table S4.

## Discussion

In a Chinese general population, part of the Nelcin-B3 project, over one-third of participants had lung nodules ≥ 30.0 mm^3^ detected by LDCT, with individuals who never smoked showing a slightly lower prevalence than those who smoke(d). A substantial difference was found in the nodule count per person between individuals who never smoked and who smoke(d). However, interestingly, no significant differences were observed in various imaging features, including nodule size, location, shape, edge, attachment type, calcification, and PFNs.

In our study, solid and subsolid nodules were identified in 33.6% and 4.3% of participants, respectively. Compared with other general population studies, the prevalence of solid nodules in our cohort was lower than that reported in the Netherlands (41.8%) [18], higher than in South Korea (16.2%) [19], and similar to that reported in Turkey (30.5%) [20] and Shanghai (29.9%) [21]. Additionally, the subsolid nodule prevalence in our study population exceeded that reported in a Dutch general population (2%) [8], but was lower than in Turkey (17.2%) [20], South Korea (9.1%) [22] and Taiwan (16.3%) [10]. Beyond inherent racial differences (e.g., Asian vs. Caucasian), the variation in nodule prevalence may also stem from differences in other population characteristics (e.g., age, sex, and smoking status profile) and the detection threshold of lung nodules. For instance, both our study and the Dutch study used a volume threshold of 30.0 mm^3^ (approximately 3.9 mm) [8], studies in Turkey and South Korea used a 4 mm diameter cutoff [19, 20, 23], and studies in Shanghai and Taiwan included nodules of all sizes [10, 21]. In addition, consistent with several previous studies [13, 18, 19], we found that individuals who never smoked had a significantly lower lung nodule prevalence compared to those who smoke(d) (33.6% vs. 42.4%). This finding is likely explained by smoking-induced chronic inflammation in the lung parenchyma, which can promote the formation of lung nodules [24].

Understanding nodule count distribution by smoking status is crucial for risk stratification, given that smoking is the primary risk factor for lung cancer. In the NELSON trial, which focused on a high-risk heavy smoking population (median pack-year: 38 [IQR 30–50]), malignancy risk increased with nodule count from one to four but decreased in participants with ≥ 5 nodules [25]. In contrast, the PanCan study, also targeting high-risk smokers (median pack-year: 50 [IQR 41–62]), demonstrated that having multiple nodules was associated with a lower risk of cancer compared to a single nodule [26]. Our general Chinese population-based study (median pack-year: 20 [IQR 12–31]) showed that individuals who never smoked more often had a single nodule, while individuals who smoke(d) more frequently had two nodules, which is in line with findings from the NELSON trial. However, a Dutch general population study by Cai et al reported no such difference between individuals who never smoked and who smoke(d) (two nodules: 21.7% vs. 22.0%–22.1%). This discrepancy may be explained by the difference in smoking intensity (defined as smoking exposure in pack-years for individuals who smoke[d]); our cohort had a higher median pack-year history (20 vs. 7.6–15.9 years in Cai et al). Regarding higher nodule counts, in our cohort, no significant difference was observed in the prevalence of ≥ 5 nodules between individuals who never smoked and who smoke(d). This finding contrasts with the malignancy-nodule count relationship in both the NELSON and PanCan studies, but aligns more closely with the study by Cai et al (≥ 5 nodules: 4.5% in individuals who never smoked vs. 4.2%–8.5% in those who smoke[d]) [8], also suggesting that smoking intensity may influence the association between nodule count and malignancy risk. Overall, these comparisons underscore that the distribution of nodule count and their associated malignancy risk could be highly dependent on the underlying smoking intensity of the study population.

Regarding nodule CT features, our study found no significant differences in nodule size, location, shape, edge, attachment, calcification, and the presence of PFN between smoking groups. Based on prior evidence, larger nodule size, upper lobe location, and irregular or spiculated edges are considered as high-risk features associated with a higher risk of lung cancer, whereas specific nodule calcification and PFNs are generally linked to benignity [4, 5, 7, 26–29]. Given the well-established link between smoking and lung cancer, we expected a higher prevalence of these high-risk imaging features and a lower prevalence of these low-risk imaging features among individuals who smoke(d). However, this expected pattern was not observed in our data. These unexpected findings may again be attributed to the relatively low intensity of smoking exposure in our general population, compared with lung screening studies that primarily included heavy smokers. Our findings are consistent with those from other general population studies. For example, in a Korean general population (48.0% individuals who never smoked), Kim et al found no significant difference in nodule size between individuals who never smoked and who smoke(d) (7.1 mm vs. 6.9 mm, *p* = 0.24) [19]. Similarly, in a general Dutch population, Cai et al reported that CT features, including nodule size, location, attachment, calcification, and PFN, did not differ significantly between smoking groups [8].

Although the overall association between nodule CT features and smoking status observed in our study is broadly consistent with that reported in the Western cohort [8], the implications differ in the Asian context due to substantial differences in lung cancer epidemiology. In East Asian populations, approximately 30% of lung cancers occur in individuals who never smoked, much higher than that in Western populations (10–15%) [30]. Therefore, relying solely on smoking history may be insufficient to characterize malignancy-associated imaging patterns and may consequently underestimate nodule malignancy risk in Asian populations. Beyond smoking intensity, other factors relevant to Asian populations may influence pulmonary nodule characteristics. Several studies have suggested that both genetic factors (e.g., EGFR mutation) and environmental exposures (e.g., passive smoking, indoor cooking oil fumes, and outdoor air pollution) may contribute significantly to lung cancer risk in Asian individuals who never smoked [12, 31]. These factors may also affect lung nodule development and morphology, potentially mimicking imaging features typically associated with smoking. Furthermore, geographical differences in endemic pulmonary infection also warrant consideration. China, for instance, has a higher incidence of tuberculosis compared to Europe (59.2 vs. < 10 per 100,000 population) [11]. Given that smokers are more susceptible to tuberculosis infection [32, 33], post-infectious changes may lead to more benign nodules in smokers, thereby potentially reducing the proportion of malignancy-associated features at the per-participant level.

This study has several limitations. First, LDCT scans were interpreted independently by one of three radiologists, without consensus reading. Although all readers received standardized training in lung nodule assessment and semi-automated size measurement to minimize variability, residual interobserver variability in determining nodule CT features (size, density type, and morphology) remains a limitation. However, the added value of consensus double reading in reducing such variability was found to be limited at baseline screening [34]. Second, we did not have access to pathological diagnostic data for lung cancer. This limited our ability to directly compare malignant and benign nodules and to assess the clinical relevance of CT features in predicting malignancy. Future studies incorporating pathologically confirmed outcomes are warranted to validate the observed associations between smoking status and CT imaging features, thereby enhancing the clinical relevance of our findings. Third, our cohort included only a small number of female smokers. However, the prevalence of smoking exposure among women is generally very low in Asian cohorts (0.4–17.5%) [35], ensuring that findings remain representative. Fourth, some nodule features (e.g., nodule volume > 250 mm^3^, location in lobe, calcification) showed notable association with smoking status but did not reach statistical significance. Post hoc power analysis suggested that our sample size may be insufficient to detect moderate differences, highlighting the need for validation in larger cohorts. Finally, subgroup analyses were limited by the relatively small number of individuals who formerly smoked with pulmonary nodules (*n* = 91). Thus, individuals who currently smoke and who formerly smoked were combined into a single “individuals who smoke(d)” group. However, multilevel analysis, when performed again without individuals who formerly smoked, did not affect the final results (Table S4).

In conclusion, this study in a Chinese general population revealed that, while smoking status was associated with slightly higher lung nodule prevalence, key nodule CT features did not show significant differences between individuals who never smoked and who smoke(d). This finding suggested that in Asian populations, the radiological appearance of lung nodules may be influenced by risk factors beyond smoking, potentially including a high incidence of tuberculosis and other environmental or genetic factors. These observations underscore the need for population-specific risk stratification strategies in lung nodule assessment and call for further research with a larger sample size to evaluate the influence of non-smoking-related factors on nodule characteristics and, ultimately, lung cancer risk in Asian populations.

## Supplementary information

ELECTRONIC SUPPLEMENTARY MATERIAL

## Abbreviations

aOR: Adjusted odds ratio
CI: Confidence interval
COPD: Chronic obstructive pulmonary disease
CT: Computed tomography
IQR: Interquartile range
LDCT: Low-dose CT
NELCIN-B3: Netherlands and China Big 3 diseases
PFN: Perifissural nodule

## References

[1] Gould MK, Tang T, Liu I-LA et al (2015) Recent trends in the identification of incidental pulmonary nodules. Am J Respir Crit Care Med 192:1208–1214. 10.1164/rccm.201505-0990OC 26214244 10.1164/rccm.201505-0990OC

[2] Hendrix W, Rutten M, Hendrix N et al (2023) Trends in the incidence of pulmonary nodules in chest computed tomography: 10-year results from two Dutch hospitals. Eur Radiol 33:8279–8288. 10.1007/s00330-023-09826-3 37338552 10.1007/s00330-023-09826-3 PMC10598118

[3] Anderson IJ, Davis AM (2018) Incidental pulmonary nodules detected on CT images. JAMA 320:2260. 10.1001/jama.2018.16336 30419095 10.1001/jama.2018.16336

[4] de Hoop B, van Ginneken B, Gietema H, Prokop M (2012) Pulmonary perifissural nodules on CT scans: rapid growth is not a predictor of malignancy. Radiology 265:611–616. 10.1148/radiol.12112351 22929331 10.1148/radiol.12112351

[5] Horeweg N, van der Aalst CM, Thunnissen E et al (2013) Characteristics of lung cancers detected by computer tomography screening in the randomized NELSON trial. Am J Respir Crit Care Med 187:848–854. 10.1164/rccm.201209-1651OC 23348977 10.1164/rccm.201209-1651OC

[6] Horeweg N, van Rosmalen J, Heuvelmans MA et al (2014) Lung cancer probability in patients with CT-detected pulmonary nodules: a prespecified analysis of data from the NELSON trial of low-dose CT screening. Lancet Oncol 15:1332–1341. 10.1016/S1470-2045(14)70389-4 25282285 10.1016/S1470-2045(14)70389-4

[7] MacMahon H, Naidich DP, Goo JM et al (2017) Guidelines for management of incidental pulmonary nodules detected on CT images: from the Fleischner Society 2017. Radiology 284:228–243. 10.1148/radiol.2017161659 28240562 10.1148/radiol.2017161659

[8] Cai J, Vonder M, Heuvelmans MA et al (2022) CT characteristics of solid pulmonary nodules of never smokers versus smokers: a population-based study. Eur J Radiol 154:110410. 10.1016/j.ejrad.2022.110410 35777080 10.1016/j.ejrad.2022.110410

[9] Thun MJ, Hannan LM, Adams-Campbell LL et al (2008) Lung cancer occurrence in never-smokers: an analysis of 13 cohorts and 22 cancer registry studies. PLoS Med 5:e185. 10.1371/journal.pmed.0050185 18788891 10.1371/journal.pmed.0050185 PMC2531137

[10] Chang Y-C, Hung Y-C, Wu Y-J et al (2025) Understanding East-West differences in subsolid nodules: prevalence and overdiagnosis implications in lung cancer screening. Ann Med 57:2478321. 10.1080/07853890.2025.2478321 40075292 10.1080/07853890.2025.2478321 PMC11912254

[11] World Health Organization (2024) Global tuberculosis report 2024. Available via https://www.who.int/teams/global-programme-on-tuberculosis-and-lung-health/tb-reports/global-tuberculosis-report-2024. Accessed 1 July 2025

[12] Chang G-C, Chiu C-H, Yu C-J et al (2024) Low-dose CT screening among never-smokers with or without a family history of lung cancer in Taiwan: a prospective cohort study. Lancet Respir Med 12:141–152. 10.1016/S2213-2600(23)00338-7 38042167 10.1016/S2213-2600(23)00338-7

[13] Kang H-R, Cho JY, Lee SH et al (2019) Role of low-dose computerized tomography in lung cancer screening among never-smokers. J Thorac Oncol 14:436–444. 10.1016/j.jtho.2018.11.002 30445189 10.1016/j.jtho.2018.11.002

[14] Li F, Sone S, Abe H et al (2003) Low-dose computed tomography screening for lung cancer in a general population. Acad Radiol 10:1013–1020. 10.1016/S1076-6332(03)00150-8 13678090 10.1016/s1076-6332(03)00150-8

[15] Yihui D, Yingru Z, Grigory S et al (2019) Methods of computed tomography screening and management of lung cancer in Tianjin: design of a population-based cohort study. Cancer Biol Med 16:181. 10.20892/j.issn.2095-3941.2018.0237 31119059 10.20892/j.issn.2095-3941.2018.0237 PMC6528449

[16] Li Y, Du Y, Huang Y et al (2021) Community-based lung cancer screening by low-dose computed tomography in China: first round results and a meta-analysis. Eur J Radiol 144:109988. 10.1016/j.ejrad.2021.109988 34695695 10.1016/j.ejrad.2021.109988

[17] Du Y, Li Q, Sidorenkov G et al (2021) Computed tomography screening for early lung cancer, COPD and cardiovascular disease in Shanghai: rationale and design of a population-based comparative study. Acad Radiol 28:36–45. 10.1016/j.acra.2020.01.020 32151538 10.1016/j.acra.2020.01.020

[18] Cai J, Vonder M, Du Y et al (2024) Who is at risk of lung nodules on low-dose CT in a Western country? A population-based approach. Eur Respir J 63:2301736. 10.1183/13993003.01736-2023 38697647 10.1183/13993003.01736-2023 PMC11154756

[19] Kim YW, Kang H-R, Kwon BS et al (2020) Low-dose chest computed tomographic screening and invasive diagnosis of pulmonary nodules for lung cancer in never-smokers. Eur Respir J 56:2000177. 10.1183/13993003.00177-2020 32482786 10.1183/13993003.00177-2020

[20] Ogan N, Baha A, Özan Sanhal E et al (2019) Incidental pulmonary nodule frequency in Turkey. Tuberk Toraks 67:190–196. 10.5578/tt.68532 31709950 10.5578/tt.68532

[21] Fan L, Wang Y, Zhou Y et al (2019) Lung cancer screening with low-dose CT: baseline screening results in Shanghai. Acad Radiol 26:1283–1291. 10.1016/j.acra.2018.12.002 30554839 10.1016/j.acra.2018.12.002

[22] Kim YW, Kwon BS, Lim SY et al (2021) Lung cancer probability and clinical outcomes of baseline and new subsolid nodules detected on low-dose CT screening. Thorax 76:980–988. 10.1136/thoraxjnl-2020-215107 33859050 10.1136/thoraxjnl-2020-215107 PMC8461405

[23] Kakinuma R, Muramatsu Y, Asamura H et al (2020) Low-dose CT lung cancer screening in never-smokers and smokers: results of an eight-year observational study. Transl Lung Cancer Res 9:10–22. 10.21037/tlcr.2020.01.13 32206549 10.21037/tlcr.2020.01.13 PMC7082286

[24] Lederer DJ, Enright PL, Kawut SM et al (2009) Cigarette smoking is associated with subclinical parenchymal lung disease: the Multi-Ethnic Study of Atherosclerosis (MESA)-lung study. Am J Respir Crit Care Med 180:407–414. 10.1164/rccm.200812-1966OC 19542480 10.1164/rccm.200812-1966OC PMC2742759

[25] Heuvelmans MA, Walter JE, Peters RB et al (2017) Relationship between nodule count and lung cancer probability in baseline CT lung cancer screening: the NELSON study. Lung Cancer 113:45–50. 10.1016/j.lungcan.2017.08.023 29110848 10.1016/j.lungcan.2017.08.023

[26] McWilliams A, Tammemagi MC, Mayo JR et al (2013) Probability of cancer in pulmonary nodules detected on first screening CT. N Engl J Med 369:910–919. 10.1056/NEJMoa1214726 24004118 10.1056/NEJMoa1214726 PMC3951177

[27] Lindell RM, Hartman TE, Swensen SJ et al (2007) Five-year lung cancer screening experience: CT appearance, growth rate, location, and histologic features of 61 lung cancers. Radiology 242:555–562. 10.1148/radiol.2422052090 17255425 10.1148/radiol.2422052090

[28] Xu DM, Van Der Zaag-Loonen HJ, Oudkerk M et al (2009) Smooth or attached solid indeterminate nodules detected at baseline CT screening in the NELSON study: cancer risk during 1 year of follow-up. Radiology 250:264–272. 10.1148/radiol.2493070847 18984780 10.1148/radiol.2493070847

[29] Christensen J, Prosper AE, Wu CC et al (2024) ACR Lung-RADS v2022: assessment categories and management recommendations. J Am Coll Radiol 21:473–488. 10.1016/j.jacr.2023.09.009 37820837 10.1016/j.jacr.2023.09.009

[30] Zhou F, Zhou C (2018) Lung cancer in never smokers—the East Asian experience. Transl Lung Cancer Res 7:450–463. 10.21037/tlcr.2018.05.14 30225210 10.21037/tlcr.2018.05.14 PMC6131183

[31] Lam DC-L, Liam C-K, Andarini S et al (2023) Lung cancer screening in Asia: an expert consensus report. J Thorac Oncol 18:1303–1322. 10.1016/j.jtho.2023.06.014 37390982 10.1016/j.jtho.2023.06.014

[32] Quan DH, Kwong AJ, Hansbro PM, Britton WJ (2022) No smoke without fire: the impact of cigarette smoking on the immune control of tuberculosis. Eur Respir Rev 31:210252. 10.1183/16000617.0252-2021 35675921 10.1183/16000617.0252-2021 PMC9488690

[33] Den Boon S (2005) Association between smoking and tuberculosis infection: a population survey in a high tuberculosis incidence area. Thorax 60:555–557. 10.1136/thx.2004.030924 15994262 10.1136/thx.2004.030924 PMC1747449

[34] Wang Y, van Klaveren RJ, de Bock GH et al (2012) No benefit for consensus double reading at baseline screening for lung cancer with the use of semiautomated volumetry software. Radiology 262:320–326. 10.1148/radiol.11102289 22106357 10.1148/radiol.11102289

[35] Yang JJ, Yu D, Wen W et al (2019) Tobacco smoking and mortality in Asia: a pooled meta-analysis. JAMA Netw Open 2:e191474. 10.1001/jamanetworkopen.2019.1474 30924901 10.1001/jamanetworkopen.2019.1474 PMC6450311

