# Supplementary material for: Features of low-dose CT-detected lung nodules: individuals who never smoked vs. who smoke(d) in a Chinese general population
Source: Eur Radiol. 2026 Apr 17;36(8):6647–56. doi: 10.1007/s00330-026-12517-4 (PMC13341849; doi:10.1007/s00330-026-12517-4)

Features of Low-dose CT Detected Lung Nodules: Individuals Who Never Smoked vs. Who Smoke(d) in a Chinese General Population

ELECTRONIC SUPPLEMENTARY MATERIAL

Eur Radiol (2026) Mao YF, Dorrius MD, Sidorenkov G, et al.

Table S1. Definitions and questionnaire of clinical characteristics of participants.

Variables	Definitions	Questionnaire
Second-hand smoking	The exposure to other people's tobacco smoke for ≥15 min on ≥1 day per week indoors	In the indoor environment where you live or work for a long period of time, did you inhale smoke that others produce at least 1 day a week for more than 15 minutes?<br>(1) No □<br>(2) Yes, you live or work in this smokey indoor environment for □□ years □
**Smoking status**		
Individuals who never smoked	Individuals who had never smoked or smoked for <6 months	Have you ever sucked at least 1 cigarette a day and lasted for 6 months or more?<br>(1) never suck □ (2) Yes, is still sucking □ (3) have ever smoked but quit smoking for □□ years □<br>If you have been or are still smoking now, the average number of cigarettes daily take is □□, and the total number of years of smoking is □□ years.
Individuals who smoke(d)	Individuals who smoked ≥1 cigarette a day for ≥ 6 months, regardless of whether they quit or not before the interview. This definition included both individuals who currently smoke and who formerly smoked	

Table S2. Characteristics of 2033 included participants.

Variables		Overall (n = 2033)	Individuals who never smoked (n = 1320)	Individuals who smoke(d) (n = 713)
Age (year), median *		62 (57-66)	61 (56-65)	63 (58-66)
Sex				
	Female	1080 (53.1%)	1041 (78.9%)	39 (5.5%)
	Male	953 (46.9%)	279 (21.1%)	674 (94.5%)
Pack-years, median (IQR) *		-	-	20 (10-30)
Pack-years				
	< 20	1603 (78.8%)	1320 (100%)	283 (39.7%)
	≥ 20	415 (20.4%)	0	415 (58.2%)
	Missing	15 (0.7%)	0	15 (2.1%)
Second-hand smoking (years)				
	No	1148 (56.5%)	860 (65.2%)	288 (40.4%)
	Yes, < 10	46 (2.3%)	28 (2.1%)	18 (2.5%)
	Yes, ≥ 10	830 (40.8%)	426 (32.3%)	404 (56.7%)
	Missing	9 (0.4%)	6 (0.5%)	3 (0.4%)
Lung nodule present				
	Yes	746 (36.7%)	444 (33.6%)	302 (42.4%)
	No	1287 (62.3%)	876 (66.4%)	411 (57.6%)

* Non-normally distributed data are medians with interquartile ranges in parentheses.

Table S3. Association between smoking status and nodule CT features (univariate multilevel analyses adjusted for age, sex and second-hand smoking exposure).

Variables	Individuals who never smoked compared to individuals who smoke(d)	
	Adjusted OR (95% CI) *	*p*-value
**Nodule size**		
Average diameter, mm		
< 6	1 (ref)	
6-8	0.99 (0.57-1.71)	0.98
> 8	1.24 (0.62-2.50)	0.55
Total volume, mm^3		
< 100	1 (ref)	
100-250	1.00 (0.54-1.84)	0.99
> 250	1.73 (0.71-4.21)	0.23
**Nodule location**		
Location within lung		
Central	1 (ref)	
Peripheral	1.31 (0.68-2.58)	0.44
Location in lobe		
Upper lobe	1 (ref)	
Lower/middle lobe	1.40 (0.84-2.31)	0.19
**Nodule shape**		
Regular	1 (ref)	
Irregular	1.40 (0.59-3.34)	0.45
**Nodule edge**		
Smooth	1 (ref)	
Non-smooth	1.06 (0.53-2.11)	0.87
**Nodule calcification**		
No	1 (ref)	
Yes	0.55 (0.29-1.05)	0.07
**Perifissural nodule (PFN)**		
No	1 (ref)	
Typical/atypical PFN	0.98 (0.54-1.77)	0.95
**Nodule attachment**		
Intraparenchymal	1 (ref)	
Pleura-attached	1.15 (0.63-2.09)	0.65
Fissure-attached	1.20 (0.62-2.35)	0.59
Vessel-attached	1.05 (0.53-2.08)	0.89

OR: odds ratio. 95% CI: confidence interval.
* Odd ratio is adjusted by age, sex and second-hand smoking (categorical data: <10 or ≥10 years).

Table S4. Association between smoking status (excluding individuals who formerly smoked) and nodule CT features (univariate multilevel analyses adjusted for age, and sex).

Variables	Individuals who never smoked compared to individuals who currently smoke	
	Adjusted OR (95% CI) *	*p*-value
**Nodule size**		
Average diameter, mm		
< 6	1 (ref)	
6-8	0.92 (0.53-1.63)	0.78
> 8	1.31 (0.62-2.78)	0.48
Total volume, mm^3		
< 100	1 (ref)	
100-250	1.17 (0.61-2.23)	0.64
> 250	1.68 (0.66-4.27)	0.28
**Nodule location**		
Location within lung		
Central	1 (ref)	
Peripheral	1.51 (0.75-3.02)	0.25
Location in lobe		
Upper lobe	1 (ref)	
Lower/middle lobe	1.49 (0.88-2.53)	0.14
**Nodule shape**		
Regular	1 (ref)	
Irregular	1.52 (0.60-3.87)	0.38
**Nodule edge**		
Smooth	1 (ref)	
Non-smooth	1.08 (0.52-2.21)	0.84
**Nodule calcification**		
No	1 (ref)	
Yes	0.58 (0.29-1.15)	0.12
**Perifissural nodule (PFN)**		
No	1 (ref)	
Typical/atypical PFN	1.04 (0.56-1.96)	0.89
**Nodule attachment**		
Intraparenchymal	1 (ref)	
Pleura-attached	0.96 (0.52-1.79)	0.89
Fissure-attached	1.20 (0.58-2.48)	0.62
Vessel-attached	1.01 (0.50-2.05)	0.97

OR: odds ratio. 95% CI: confidence interval.
* Odd ratio is adjusted by age, and sex.

Supplementary Appendix 1. Statistical analysis for the lung nodule prevalence.

The prevalence of lung nodules (≥ 30 mm^3) was stratified by age in our study population. Differences in nodule prevalence between individuals who never smoked and who smoke(d), or between males and females across age categories, were evaluated by Chi-square test. Correlations between age and lung nodule prevalence were evaluated by Spearman rank correlation coefficient (ρ), according to the following cut-offs: $\rho < 0.20$, very weak or negligible; $\rho = 0.20–0.39$, weak or low; $\rho = 0.40–0.70$, moderate; and $\rho > 0.70$, strong or high.

Supplementary Appendix 2. Results for the lung nodule prevalence per age category.

At baseline LDCT screening, 746 (36.7%) participants had at least one lung nodule (≥ 30.0 mm^3), including 690 (33.9%) with at least one solid nodules (≥ 30.0 mm^3), and 87 (4.3%) with at least one subsolid (≥ 30.0 mm^3), respectively. Overall nodule prevalence was higher in individuals who smoke(d) than in those who never smoked (42.4% [302/713] vs. 33.6% [444/1320], $p < 0.001$). The higher nodule prevalence in individuals who smoke(d) was observed across most age subgroups, with a statistically significant difference evident only in the 70-74-years group ($p < 0.001$). An age-related increase in the prevalence of lung nodules was observed in both individuals who never smoked ($\rho = 0.095$, $p < 0.001$) and who smoke(d) ($\rho = 0.23$, $p < 0.001$).

Male consistently showed a higher prevalence of lung nodules across most age subgroups (range: 25.0%-54.6%), compared to females (range: 24.0%-39.6%). An exception was observed in the 40–44.9-year subgroup, where females showed a slightly higher prevalence than males 27.8% [10/36] vs. 26.1% [6/23]). A statistically significant difference by sex was observed only in the 70-74-years group ($p = 0.004$). Nodule prevalence increased with age in both sexes, although the correlation coefficients were modest (female: $\rho = 0.079$ [$p = 0.01$]; male: $\rho = 0.18$ [$p < 0.001$]).

Figure S1. (a) prevalence of lung nodules (≥30 mm^3) between smoking status, stratified by age, and (b) prevalence of lung nodules among females and males, stratified by age.

Note: The prevalence of lung nodule (≥30 mm^3) was the sum of proportion of participants with only solid, only subsolid, and with both. The prevalence of solid nodule (≥30 mm^3) was the sum of proportion of participants with only solid and with both. The prevalence of subsolid nodule was the sum of proportion of participants with only subsolid and with both.

Abbreviation: Never, individuals who never smoked; Ever: Individuals who smoke(d); F, female; M, male; With both: individuals who had both solid and subsolid nodules identified on their LDCT scans.

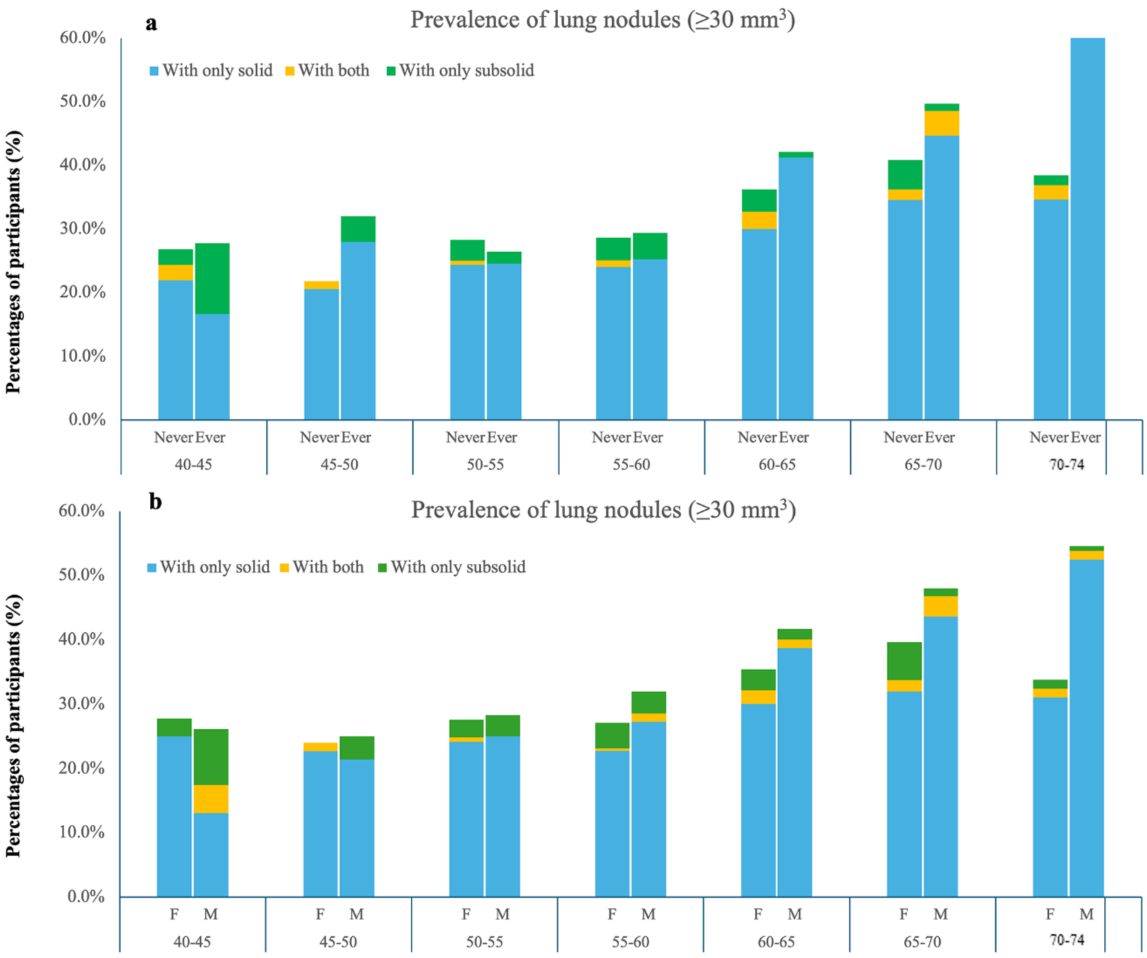

Supplement: Supplementary file 1 — ELECTRONIC SUPPLEMENTARY MATERIAL [file 330_2026_12517_MOESM1_ESM.pdf]
